# Co-ordinated stage-dependent enhancement of *Plasmodium falciparum *antioxidant enzymes and heat shock protein expression in parasites growing in oxidatively stressed or G6PD-deficient red blood cells

**DOI:** 10.1186/1475-2875-8-113

**Published:** 2009-05-29

**Authors:** Oscar Bate Akide-Ndunge, Elisa Tambini, Giuliana Giribaldi, Paul J McMillan, Sylke Müller, Paolo Arese, Francesco Turrini

**Affiliations:** 1 Department of Genetics, Biology and Biochemistry, University of Torino Medical School, Torino, Italy; 2 Division of Infection and Immunity, and Wellcome Centre for Molecular Parasitology, University of Glasgow, Glasgow, UK

## Abstract

**Background:**

*Plasmodium falciparum*-parasitized red blood cells (RBCs) are equipped with protective antioxidant enzymes and heat shock proteins (HSPs). The latter are only considered to protect against thermal stress. Important issues are poorly explored: first, it is insufficiently known how both systems are expressed in relation to the parasite developmental stage; secondly, it is unknown whether *P. falciparum *HSPs are redox-responsive, in view of redox sensitivity of HSP in eukaryotic cells; thirdly, it is poorly known how the antioxidant defense machinery would respond to increased oxidative stress or inhibited antioxidant defense. Those issues are interesting as several antimalarials increase the oxidative stress or block antioxidant defense in the parasitized RBC. In addition, numerous inhibitors of HSPs are currently developed for cancer therapy and might be tested as anti-malarials. Thus, the joint disruption of the parasite antioxidant enzymes/HSP system would interfere with parasite growth and open new perspectives for anti-malaria therapy.

**Methods:**

Stage-dependent mRNA expression of ten representative *P. falciparum *antioxidant enzymes and *hsp*60/70–2/70–3/75/90 was studied by quantitative real-time RT-PCR in parasites growing in normal RBCs, in RBCs oxidatively-stressed by moderate H2O2 generation and in G6PD-deficient RBCs. Protein expression of antioxidant enzymes was assayed by Western blotting. The pentosephosphate-pathway flux was measured in isolated parasites after Sendai-virus lysis of RBC membrane.

**Results:**

In parasites growing in normal RBCs, mRNA expression of antioxidant enzymes and HSPs displayed co-ordinated stage-dependent modulation, being low at ring, highest at early trophozoite and again very low at schizont stage. Additional exogenous oxidative stress or growth in antioxidant blunted G6PD-deficient RBCs indicated remarkable flexibility of both systems, manifested by enhanced, co-ordinated mRNA expression of antioxidant enzymes and HSPs. Protein expression of antioxidant enzymes was also increased in oxidatively-stressed trophozoites.

**Conclusion:**

Results indicated that mRNA expression of parasite antioxidant enzymes and HSPs was co-ordinated and stage-dependent. Secondly, both systems were redox-responsive and showed remarkably increased and co-ordinated expression in oxidatively-stressed parasites and in parasites growing in antioxidant blunted G6PD-deficient RBCs. Lastly, as important anti-malarials either increase oxidant stress or impair antioxidant defense, results may encourage the inclusion of anti-HSP molecules in anti-malarial combined drugs.

## Background

During the intraerythrocytic development of *Plasmodium falciparum*, reactive oxygen species (ROS) are produced by both the parasite and the host red blood cells (RBCs) [1,2]. To cope with ROS and maintain a redox equilibrium, the parasites are equipped with effective scavengers, such as reduced glutathione (GSH) [3-5], thioredoxins [6], and protective antioxidant enzymes present in the cytosol, mitochondrion or apicoplast that directly inactivate ROS or regenerate scavengers [7-11]. Protection of all subcellular compartments from oxidative stress is important and the biosynthesis of [Fe-S] clusters in both apicoplast and mitochondrion is particularly prone to oxidation and needs to be protected from ROS [12]. *Plasmodium *appears to be equipped with antioxidant systems in its organelles and a superoxide dismutase and a functional peroxiredoxin system are localized in the mitochondrial compartment [10,11].

In addition to antioxidant enzymes the parasite is equipped with a number of heat shock proteins (HSPs) [13,14] that form a second protective system against stress present in *falciparum *malaria, notably against thermal stress typical for this disease. HSPs are highly conserved and universally present molecular chaperones that protect cell structures and organelles against thermal, chemical and redox stress. In addition, HSPs play crucial roles in folding/unfolding/assembly of proteins, transport/sorting of proteins into correct subcellular compartments, cell-cycle control, signaling, and antigen presentation [see ref. [15] and [16] for review]. Several HSPs are localized in the mitochondria [12,17-20] supporting the hypothesis that both cytosol and organelles are subjected to stress. Both protective systems are essential for parasite survival, and drugs that inhibit or interfere with the first or second system may lead to parasite death.

Present work addresses issues that received little attention so far: the first one is how antioxidant enzymes and HSPs are expressed in relation to the parasite development in the RBC. The second issue is whether and how *P. falciparum *HSPs take part into antioxidant defense, and are modulated by oxidative stress possibly in a co-ordinated way with the antioxidant enzyme system. Redox-sensitivity of HSPs is unexplored in *Plasmodium*, although a number of HSPs are remarkably redox-responsive in most eukaryotic cells [12,19-24]. The third issue regards how the antioxidant enzymes and HSPs respond to increased oxidative stress or inhibited antioxidant defense. Such response is of interest because the joint disruption of the antioxidant enzymes/HSP system of the parasite would interfere with parasite growth or enhance their removal by the host's phagocytes and open new perspectives of combined anti-malarial therapy. Indeed, a number of anti-malarials such as chloroquine and artemisinin, peroxidic antimalarials, anthroquinones and xanthones exert their activity, at least in part, by increasing the oxidative stress in the parasitized RBC [25-27], while others such as inhibitors of parasitic glutathione reductase aim at inhibiting antioxidant defense mechanisms [28,29]. In addition, widespread genetic RBC defects protective against malaria such as glucose-6-phosphate dehydrogenase (G6PD) deficiency, sickle-cell trait and β-thalassaemia impose an additional oxidative stress upon host and parasite, and induce early removal of parasitized RBCs by the host's phagocytes [30,31]. In analogy to other eukaryotic cell systems [2,7,17-24], parasitic genes encoding antioxidant enzymes and HSPs may upmodulate their responses, and thus reduce efficacy of redox-active drugs. Inhibition of antioxidant enzymes and HSPs may thus represent a way to kill the parasites or to enhance their phagocytic removal.

In the present study, stage-dependent mRNA expression of representative cytosolic and mitochondrial antioxidant enzymes and HSPs was assessed by quantitative real-time RT-PCR (qRT-PCR) in *P. falciparum *growing in normal, unstressed RBCs, in oxidatively stressed RBCs, and in G6PD-deficient RBCs that are characterized by blunted antioxidant defense. The study parameters included ten *Pf-*antioxidant enzymes: glutathione peroxidase-like thioredoxin peroxidase (GPx-like TPx), glutathione reductase (GR), glutathione S-transferase (GST), glutaredoxin-1 (Grx), thioredoxindependent peroxidases (1-Cys-Prx, 2-Cys-Prx), thioredoxin reductase (TR), superoxide dismutases-1 and -2 (SOD-1, SOD-2), G6PD; a glycolytic enzyme, glyceraldehyde-3-phosphate dehydrogenase (GAPDH); and a panel of five *Pf-*HSPs: HSP60, HSP70–2, HSP70–3, HSP75 and HSP90. By analogy with other eukaryotic cells, these genes/proteins are likely to respond to oxidant stress [20-24].

Results indicate that mRNA expression of both parasite antioxidant enzymes and HSPs was strictly co-ordinated and stage-dependent attaining highest values at early trophozoite stage and declining afterwards; secondly, in parasites developing in oxidatively stressed normal RBCs and in G6PD-deficient RBCs, characterized by blunted antioxidant defense, both systems were redox-responsive and showed increased mRNA and protein expression. As important anti-malarials either increase oxidant stress or decrease antioxidant defense, results may encourage the inclusion of anti-HSPs molecules in combined anti-malarial drugs.

## Methods

### Materials

Buffers, RPMI 1640 culture medium containing Hepes, methionine-free RPMI 1640, mannitol, octylphenyl-polyethylene glycol (IGEPAL CA-630), gentamicin, xanthine (X), xanthine oxidase (XO), superoxide dismutase (SOD), allopurinol and SYBR Green 1 were from Sigma, St. Louis, Missouri, USA; Percoll was from Pharmacia, Uppsala, Sweden; RLN lysis buffer was prepared according to RNeasy Mini Handbook, Qiagen, Milano, Italy; DNA-*free*™ DNase Treatment & Removal Reagents was from Ambion, Austin, Texas, USA; Tran^35^S-label was from MP Biochemicals, Irvine, CA; D- [1-^14^C] glucose was from Amersham International, Amersham, UK; Diff-Quik parasite stain was from Baxter Dade AG, Dudingen, Switzerland; Standard RNA Releaser was from Nurex, Sassari, Italy; RNaseOut RibonucleaseInhibitor, M-MLV reverse transcriptase, oligo-dT and Platinum Taq DNA polymerase were from Invitrogen, Milano, Italy; sterile plastics were from Falcon, Becton Dickinson Labware, Franklin Lakes, NJ; nitrocellulose transfer membranes were from Whatman-Schleicher & Schuell, Dassel, Germany; West Pico Chemiluminescent Substrate was from Pierce, Rockford, Illinois, USA. Bradford reagent was from Bio-Rad, Hercules, California, USA. All other reagents were purchased from common commercial sources.

### *Plasmodium falciparum *cultivation and isolation of parasitized RBCs

Venous peripheral blood samples were collected from haematologically healthy subjects and from hemizygous male subjects with the Mediterranean G6PD variant (residual G6PD activity <5%). In all cases, informed consent was obtained. Blood anticoagulated with citrate-phosphate-dextrose with adenine (CPDA-1) was kept for 12 to 24 h at 4°C and used for parasite growth experiments, metabolic and mRNA studies. Normal and G6PD-deficient RBCs were isolated from plasma and white blood cells by 80% Percoll gradient centrifugation and three washes in wash medium (RPMI 1640 containing 25 mM HEPES). For parasite growth experiments, metabolic studies and mRNA studies, *P. falciparum *parasites (Palo Alto strain, *Mycoplasma-*free) were cultivated at 2% haematocrit and synchronized as already described [30-32]. Briefly, normal schizont stage parasitized RBCs separated on mannitol containing (6% w/v) 40/80% discontinuous Percoll gradient (parasitaemia >95%) were mixed with normal or G6PDdeficient RBCs suspended in growth medium (RPMI 1640 medium containing 25 mM Hepes, 30 mM glucose, 2 mM glutamine, 2 mM adenine, 24 mM NaHCO3, 32 mg/l gentamicin, and 10% AB or A human serum, pH 7.4) to start reinfection (time 0). After 6 h incubation, cultures were separated on Percoll 40/80/90% discontinuous gradient and haemozoin and schizonts eliminated. The parasite ring stages (parasitaemia approx. 20%) were collected from the 80–90% interface, washed three times in wash medium, resuspended in growth medium and incubated for a further 4 h. At this time (10 h after reinfection), cultures were supplemented or not with xanthine oxidase (XO)/xanthine (X) and allopurinol (A) in various combinations (see below). Parasite aliquots were collected at 13 (early ring stage), 16 (late ring stage), 19 (early trophozoite stage), 28 (late trophozoite stage) and 46 (schizont stage) h after reinfection. To assess parasitaemia, slides were prepared from cultures, stained with Diff-Quik parasite stain, and 400 to 1,000 cells examined microscopically. Significance of differences in parasitaemia between control, XO/X, XO/X+A and G6PD-deficient parasitized RBCs was assessed by *t*-test for paired samples.

### Exogenous oxidative stress by XO-X treatment

Oxidative stress in form of continuous extracellular generation of hydrogen peroxide, was provided by supplementing cultures with low XO/X (XO [EC 1.1.3.22 from bovine milk, 1–2 U/mg protein] 0.1 mU/ml; X, 1 mM), or high XO/X (XO, 1 mU/ml; X, 1 mM) as indicated [24,25]. SOD (EC 1.15.1.1 from bovine RBCs, 2,500–7,000 U/mg protein) was added throughout at 100 U/ml to enhance formation of hydrogen peroxide from superoxide radical anions generated by the XO-catalyzed oxidation of X [33]. Low and high XO/X resulted in a constant steady-state levels of approx. 0.44 μM and approx. 4.4 μM hydrogen peroxide in the parasite culture, respectively [34]. Allopurinol (A) (50 μM), a scavenger of ROS and inhibitor of XO, was added or not as indicated [35].

### [^35^S]-biosynthetic labelling of *P. falciparum *proteins

For protein labelling of synchronous parasites, asynchronous *P. falciparum *culture separations were carried out on Percoll 40/80% discontinuous gradient. Schizont stage parasites were washed once in methionine-free RPMI 1640 and reinfection carried out in methionine-free RPMI 1640 supplemented with 10% human serum. At 10 h after reinfection, cultures were treated or not for 13 h with high/low XO/X with/without A. After incubation, cultures were resuspended in 0.5 ml of the same medium supplemented with Tran^35^S-label at 50 μCi/ml and incubated for 3 h at 37°C. After incubation, cultures were washed once in ice-cold PBS, 2 ml of ice-cold 20% (w/v) trichloroacetic acid (TCA) were added and the mixture was placed for 10 min on ice. Protein precipitates were washed once with 10 ml of cold 20% TCA and 5 ml of cold 10% TCA on a nitrocellulose filter paper (type GS, pore size 0.22 μm, Millipore) and air-dried. Protein concentration was determined using the Bradford method (Bio-Rad). Radioactivity was measured as counts/min (cpm)/mg protein in a liquid scintillation counter.

### Extraction of *P. falciparum *total RNA

Total RNA was extracted at 13, 16, 19, 28 and 46 h after reinfection from synchronized cultures of parasitized normal RBCs, parasitized normal RBCs treated with low XO/X and parasitized G6PD-deficient RBCs. Before the extraction of total RNA, 200 μl of parasitized RBCs (18–20% parasitaemia) were lysed in 200 μl RLN Buffer (50 mM Tris-HCl, pH 8.0, 140 mM NaCl, 1.5 mM MgCl2, 0.5% (v/v) octylphenyl-polyethylene glycol (IGEPAL CA-630) and 1000 U/ml of RNAse inhibitor) and incubated for 5 min on ice. Lysates were centrifuged at 300 × g for 5 min at 4°C. Supernatants were collected and pellets discarded. RNA was extracted from the supernatants using Standard RNA Releaser (Nurex) and chloroform according to the manufacturer's instructions. Sample lysates with Standard RNA Releaser and chloroform were centrifuged and the upper aqueous layer carefully transferred into a new tube, taking care to avoid the region just above the interface where DNA accumulates. RNA digestion was carried out using the DNA-*free*™ DNase Treatment & Removal Reagents (Ambion) to completely remove genomic parasite DNA contamination according to the manufacturer's instructions. No traces of DNA were observed in our experiments. cDNA was prepared adding 30 μl of extracted RNA (corresponding to 18 μg RNA), to 48 μl reaction mixture containing 300 ng oligo-dT and 600 U M-MLV reverse transcriptase.

### Quantitative real-time RT-PCR of enzymes and HSPs

mRNA expression levels of ten parasite antioxidant enzymes: GPx-like TPx, GR, GST, Grx, 1-Cys-Prx, 2-Cys-Prx, TR, SOD-1, SOD-2, G6PD; one parasite glycolytic enzyme, GAPDH; and five parasite HSPs: HSP60, HSP70–2, HSP70–3, HSP75, and HSP90, were measured by qRT-PCR. All oligonucleotide sequences were identified using Beacon Designer Software (PREMIER Biosoft International, Palo Alto, CA). Primer pairs of amplicons analysed by qRT-PCR are shown in Table 1. Five μl of serial dilutions (1:10 to 1:1000) of cDNA from 200 μl of *P. falciparum *untreated control sample were used to generate a standard curve. Assays were performed on the iCycler instrument (BIORAD, Hercules, CA), in a final volume of 25 μl containing 5 μl of cDNA diluted 1:10, 200 nM primer pairs, 1.25 U of Taq Platinum DNA polymerase, 3 mM MgCl2, 200 μM dNTPs and 1.7 μl of SYBR-Green diluted 1:10,000. DNA polymerase was pre-activated for 2 min at 94°C and the amplification was performed by a 40-cycles PCR (94°C, 30 sec; 62°C, 40 sec; 72°C, 30 sec). All reactions were run in triplicate. Quantification of PCR results was assessed with the 2^-ΔΔCt ^method [36]. The relative amounts of mRNAs were calculated using *Pf*-18S rRNA as a stage-dependent internal standard [37]. All analysed transcripts exhibited high linearity amplification plots (r>0.98) and similar PCR efficiency (see Table 1), confirming that the expression of each of these genes can be directly compared to one another. The specificity of PCR results was confirmed by melting curve analysis. The melting temperatures for each amplification product are indicated in Table 1.

**Table 1:** Primer pairs of amplicons analysed by qRT-PCR

Gene Name	Gene ID (Entrez Nucleotide NCBI)	Gene ID (Plasmo DB)	(5' → 3') Primer Sequences		Prod. Tm	Ampl. size	Effic.
			Forward	Reverse	°C	(bp)	(%)

18S rRNA	M19172	MAL1_18s	CAAGGAAGTTTAAGGCAACAACAG	CATATCTTTCAATCGGTAGGAGCG	86	313	100.9

GAPDH	AF030440	PF14_0598	TGGAATTGTTGAAGGTTTAATG	CTGGAATAATGTTGGATAATGC	85.5	128	98.8

GPx	Z68200	PFL0580w	TCGATGCATGATGAAAATGGAACG	AATCTAACGGGTTTGTTTTGGGTG	82	118	100.3

GR	AF027825	PF14_0192	GTTTAGGTGGAACGTGTGTCAAC	GGTGTCAAATCCGTAATGCCTGG	83.5	110	99.3

Grx	AF276083	PFC0271c	CCCATATTGTATTAAGGCAATT	TTCTCAATGTTTTCTACATGC	79	78	100.3

GST	AY014840	PF14_0187	GCTTGGAGATAATATAGTGTTATA	CTATTCCAAGGTAGGCAAAA	78.5	86	100.2

1-Cys-Prx	AF294425	PF08_0131	GGGCTATTTTATTTAGTCACCCAC	TTGATCGTGAGATTCTTTGGAGTT	83	146	96.6

2-Cys-Prx	AB037568	PF14_0368	TGGTAGATCAGTTGAGGAAGTCTT	CACTAACACCTTCTTCTGATGGTT	85.5	131	97.4

TR	X87095	PFI1170c	ATGGTGCATGTGGATATTCAGAAG	TGTCGATGAACAGCAGATATTTCC	80	177	99.8

SOD-1	Z49819	PF08_0071	TTGTGGTGGTGAGCCTCATGG	CCCCAACCGGAACCAAAATGAC	82	84	100.1

SOD-2	AY586514	PFF1130c	TTTAACCACAATTTCTTTTGGC	CACTTCCAAAATGACCTGATG	78.7	151	98.7

Hsp60^1^	U38963	PF10_0153	CCAGTCATCCTTATTAGTTATAGC	CTATATCATGAATCAAGGCTTTTC	82.5	149	99.5

Hsp70–2^2^	M18836	PFI0875w	ATGCCAACCAATCATTGTTAAATT	ATTCGTCACTATCTACATCTTCGT	84	89	100.5

Hsp70–3^3^	AB050740	PF11_0351	TTAGTTGGTGGTATGACTAGAATG	TTAATACACCTCCTTGTATAGCAG	83.5	133	100

Hsp75^4^	M28261	PF08_0054	AATTTCCCAGGAGGTATGCCC	TTCTTCAACTGTTGGTCCACTTC	90	75	97.9

Hsp90^5^	X13014	PF07_0029	CATGACTAGCTATATGTTATCCAA	AAGAGGTATCAAATAATAACCAGA	81.5	143	100.3

^1^PlasmoDB nomenclature (PDB): hsp60; ^2 ^PDB: Heat shock protein (72 kDa); ^3^PDB: Heat shock protein hsp 70 kDa homologue (73 kDa); ^4^PDB: heat shock 70 kDa protein (75 kDa); ^5^PDB: heat shock protein 86.

### Protein expression of antioxidant enzymes

Protein expression was studied in early trophozoite stages separated 19 h after reinfection from synchronized cultures of parasitized normal RBCs untreated or treated with low XO/X. Parasites were isolated from host RBCs by Sendai-virus treatment (see below) and lysed by addition of lysis buffer (100 mM Hepes, pH 7.4, 5 mM MgCl2, 10 mM EDTA, 0.5% TritonX-100, 5 μg/ml RNAse, 1 mM PMSF, 1 mM benzamidine, 2 μg/ml leupeptin, 10 μM E-64, 2 μM 1,10-phenanthroline, 4 μM pepstatin A) followed by three cycles of freeze-thawing (dry ice/37°C) and brief sonication before centrifugation at 16,000 g at 4°C for 20 min. The supernatant was treated for 5 min on ice with 1 volume of ice-cold butanol/chloroform (3:1.2, pre-chilled to -20°C) to remove haemoglobin contamination from the protein lysate. The mixture was then centrifuged at 16,000 g at 4°C for 10 min and the protein lysate (aqueous phase) was transferred to a fresh tube. Protein concentration was determined using the Bradford method (Bio-Rad). Equal amounts of protein of non-treated control lysates and lysates of treated parasites (10 μg) were separated on a 4–12% SDS-PAGE (Invitrogen) and proteins were subsequently blotted onto nitrocellulose (Whatman-Schleicher & Schuell) using a semi-dry transfer method as per manufacturer's instructions (Bio-Rad). Equal loading and transfer efficiency was also verified by Ponceau staining of the blots. The blots were hybridized with polyclonal antibodies directed against *P. falciparum *thioredoxin-dependent peroxidases (1-Cys-Prx, 2-Cys-Prx), thioredoxin reductase, superoxide dismutases (SOD-1 and -2), and glutathione reductase. Subsequently the blots were hybridized with the secondary anti-rabbit or rat horseradish peroxidase coupled antibody (Promega) at 1:5,000 for 1 h, washed in the same buffer as before and the proteins were detected using the Super Signal West Pico Chemiluminescent Substrate according to the manufacturer's instructions (Pierce) followed by densitometry. Protein expression changes in oxidatively stressed cells vs untreated controls was expressed as -fold variation of band densitometry.

### Measurement of pentose phosphate pathway (PPP) flux by [^14^C]-CO2 production from D- [1-^14^C] glucose. Assay of G6PD activity

PPP flux was measured in trophozoites growing in normal or G6PD-deficient RBCs, treated or not for 18 h with XO-X by assessing the [^14^C]-CO2 production from D- [1-^14^C] glucose [30]. Trophozoites were treated with Sendai virus (30 μg/ml, corresponding to 800 haemagglutination units) that selectively lyses RBC without affecting the parasite membranes [30,32]. Sendai-virus treated trophozoite-parasitized RBC were suspended in RPMI 1640 (without NaHCO3, but containing 25 mM Hepes, 20 mM glucose, 1 μCi/ml D- [1-^14^C] glucose and 32 mg/l gentamicin, pH 7.4) at 5% haematocrit. After 90 min of preincubation at 37°C, [^14^C]-CO2 production was measured as indicated [30]. For G6PD activity measurements, trophozoites growing in normal or G6PD-deficient RBCs were treated with Sendai virus, washed and lysed by three cycles of freeze-thawing (dry ice/37°C). Production of [^14^C]-CO2 was expressed as μmol/10^10 ^cells/h at 37°C. G6PD activity was measured spectrophotometrically at 340 nm and 37°C in the lysate as indicated [38].

### Statistical analysis

Values are expressed as mean ± SEM unless otherwise noted. The number of experiments is indicated by *n*. Significant differences between groups were assessed by one-way ANOVA followed by the Duncan's multiple-range test or Student's *t*-test depending on the experiments. p < 0.05 was chosen as the level of significance.

## Results

To optimize maximal exogenous oxidative stress compatible with full viability of *P. falciparum*, parasites were grown in presence of varying XO activities at constant 1 mM × concentration. The highest XO activity (XO 0.1 mU/ml, resulting in a constant steady-state level of approx. 0.44 μM hydrogen peroxide in the parasite culture [34]), which did not inhibit *P. falciparum *growth and parasitic protein synthesis was used throughout. Higher XO activity (XO 1 mU/ml, resulting in a constant steady-state level of approx. 4.4 μM hydrogen peroxide in the parasite culture [34]) totally abrogated protein synthesis due to the cytotoxic effect of the treatment (Table 2).

**Table 2:** Effect of exogenous oxidative stress on protein synthesis in trophozoite stage parasitized normal RBCs

Sample	Treatment	% Inhibition
Parasitized normal RBCs	0	0
Parasitized normal RBCs	low XO/X	0
Parasitized normal RBCs	high XO/X	100

Parasites were grown at low XO (0.1 mU/ml) or high XO (1 mU/ml) activity at constant × concentration (1 mM final) as indicated (for details, see Methods). Reference values (100%) for protein synthesis: trophozoites growing in normal RBCs: 663.2 ± 17.2 (counts × 10^3^/min/mg protein ± SD, n = 3); trophozoites growing in oxidatively stressed RBCs: 780.9 ± 67.3 (same); trophozoites growing in G6PD-deficient RBCs: 503.5 ± 35.1 (same).

### Stage-dependent mRNA expression of antioxidant enzymes and HSPs in parasites growing in normal RBCs

**Figure 1:**
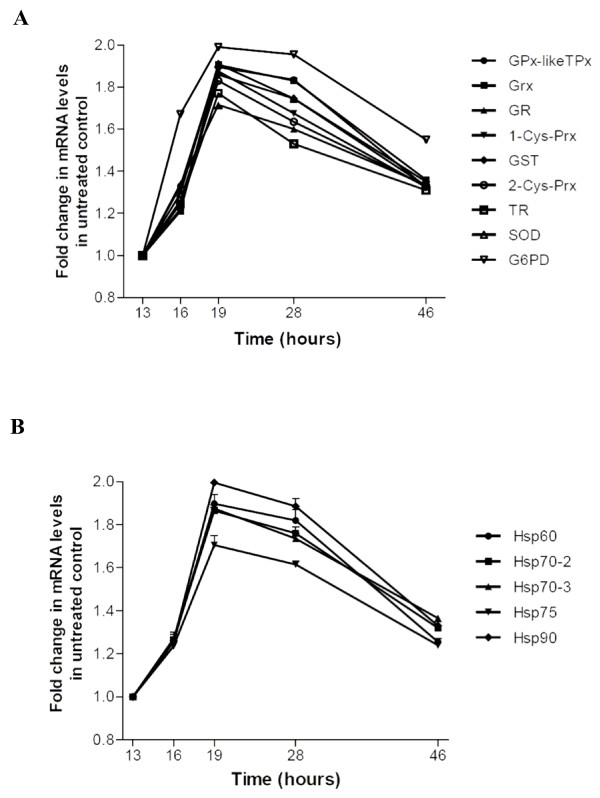
**Stage-dependent mRNA expression of parasite antioxidant enzymes (A) and Hsps (B) in parasites growing in normal RBCs**. Schizont stage parasitized normal RBCs separated on mannitol-containing discontinuous Percoll gradient (parasitaemia >95%) were mixed with normal RBCs, suspended in growth medium to start synchronous cultures (time 0). Six h after reinfection, parasite ring stages were isolated, washed three times and incubated in growth medium. At 13 (early ring stage), 16 (late ring stage), 19 (early trophozoite stage), 28 (late trophozoite stage) and 46 (schizont stage) h after reinfection, 200 μl of parasitized RBCs were lysed and RNA extracted. Changes in mRNA expression were measured using qRT-PCR. For details, see Methods. Results are presented as -fold increase over the mRNA level at early ring stage measured 13 h after reinfection using the 2^-ΔΔCt ^method. Mean values ± SEM (vertical bars), n = 1–4.

Stage-dependent mRNA expression of ten parasitic antioxidant enzymes and five HSPs was measured by qRT-PCR in *P. falciparum *growing in unstressed normal RBCs. Taking as a reference mRNA expression levels at early ring stage, a remarkably coordinated and homogenous increase in both antioxidant enzymes and HSPs expression was observed (Figure 1). All mRNA species increased approx. 1.7-2-fold from ring to early trophozoite stage at 19 h after reinfection. mRNA expression stayed high for all mRNA species considered from hour 19 to hour 28 (late trophozoite stage) and declined smoothly until the hour 46 stage (schizont stage).

### Stage-dependent mRNA expression of antioxidant enzymes, GAPDH and HSPs in parasites growing in oxidatively stressed normal RBCs

**Figure 2:**
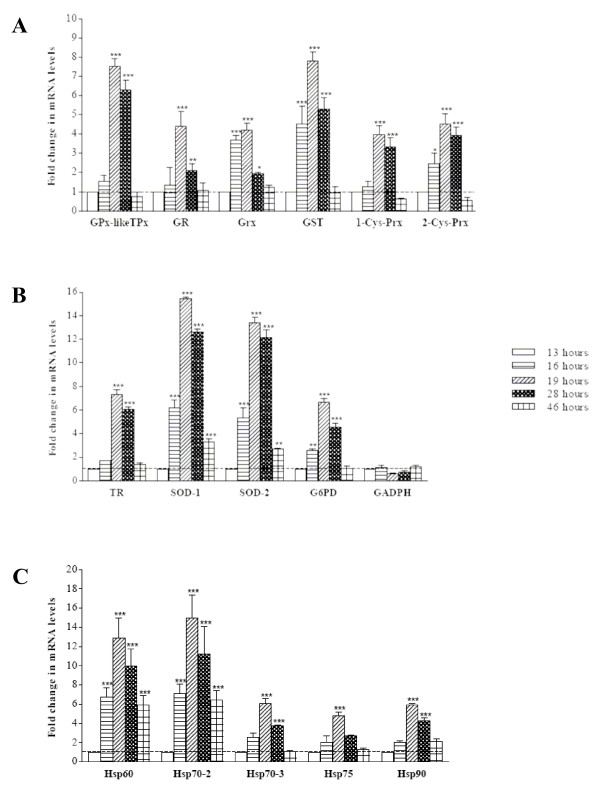
**Stage-dependent mRNA expression of parasite antioxidant enzymes and GAPDH (A; B) and Hsps (C) in parasites growing in oxidatively stressed normal RBCs**. Changes in mRNA expression were measured using qRT-PCR. For details, see legend to Figure 1 and Materials and methods. The results are presented as -fold increase over the mRNA level at early ring stage measured 13 h after reinfection using the 2^-ΔΔCt^t method. Mean values ± SEM (vertical bars), n = 5–7. Significances vs ring stage levels are indicated with symbols p < 0.001 (***), p < 0.01 (**), p < 0.05 (*).

The effect of exogenous oxidative stress exerted by XO/X treatment on the stage-dependent mRNA expression of parasitic antioxidant enzymes and HSPs is shown in Figure 2. Taking as a reference mRNA expression levels at early ring stage, all mRNA species except GAPDH were enhanced in parasites growing in oxidatively stressed normal RBCs. Remarkable enhancement in expression during the ring-to-trophozoite transition (hour 13 to hour 19) was observed in all genes except GAPDH with between 4- to 8-fold increases. Following genes were higher responders: SOD-1, 15.4-fold increase; SOD-2, 13.4-fold; HSP60, 12.9-fold; and HSP70–2, 14.9-fold. Addition of allopurinol, a specific inhibitor of XO [34], caused approx. 60% decrease of the gene expression enhancements induced by the XO/X treatment, indicating that ROS production was likely responsible for enhancement of gene expression of both antioxidant enzymes and HSPs.

### Stage-dependent mRNA expression of antioxidant enzymes and HSPs in parasites growing in G6PD-deficient RBCs

**Figure 3:**
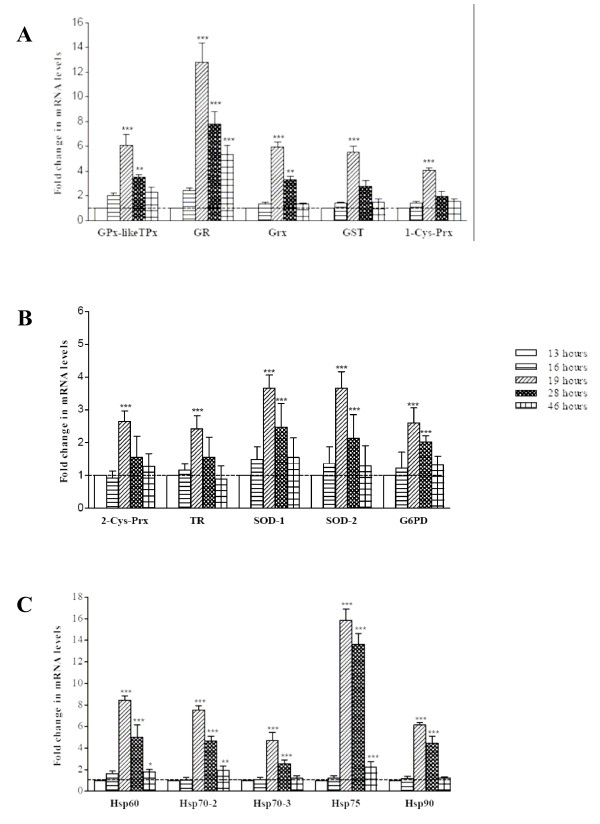
**Stage-dependent mRNA expression of parasite antioxidant enzymes (A; B) and HSPs (C) in parasites growing in G6PD-deficient (Mediterranean variant) RBCs**. Changes in mRNA expression were measured using qRT-PCR. For details, see legend to Figure 1 and Materials and methods. The results are presented as -fold increase over the mRNA level at early ring stage measured 13 h after reinfection using the 2^-ΔΔCt ^method. Mean values ± SEM, vertical bars, n = 5–7. Significances vs ring stage levels are indicated with symbols p < 0.001 (***), p < 0.01 (**), p < 0.05 (*).

The effect of parasite growth in G6PD-deficient RBCs on the stage-dependent mRNA expression of parasitic antioxidant enzymes and HSPs is shown in Figure 3. The mRNA expression pattern of parasitic antioxidant enzymes and HSPs was also modulated stage-dependently and all considered parameters significantly increased in the ring-to-trophozoite transition: GPx-like TPx (6.1-fold), GR (12.8-fold), Grx (6.0-fold), GST (5.6-fold), 1-Cys-Prx (4.1-fold), SOD-1 and SOD-2 (3.7-fold), G6PD (2.6-fold), HSP60 (8.5-fold), HSP70–2 (7.5-fold), HSP70–3 (4.7-fold), HSP75 (15.8-fold), and HSP90 (6.1-fold). The kinetics of stage-dependent mRNA expression was apparently not affected by the G6PD-deficient status of the host RBCs, as gene expression of all parameters peaked at 19 h after reinfection and declined afterwards.

**Figure 4:**
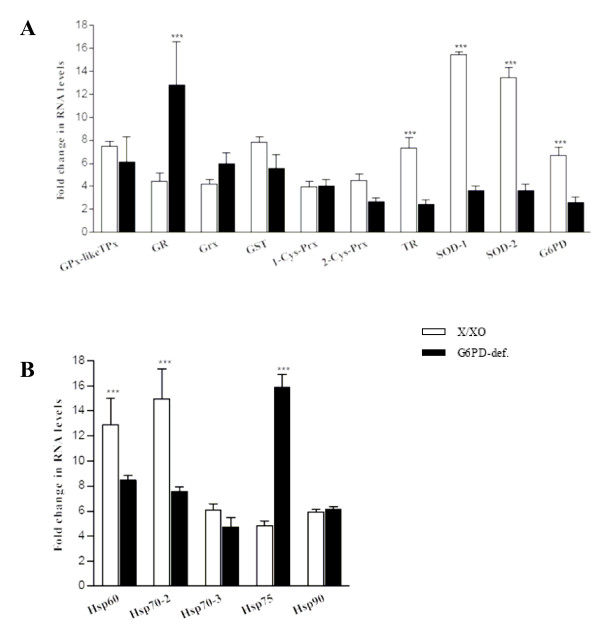
**Comparison of peak -fold mRNA expression at early trophozoite stage (parasites isolated 19 h after reinfection) in parasites growing in oxidatively stressed normal RBCs (open columns) and in G6PD-deficient RBCs (solid columns)**. Significance of stage-matched differences are indicated with symbol p < 0.001 (***). -Fold data from Figure 2 and Figure 3.

As shown in Figure 4, comparison to oxidatively-stressed parasites indicates that peak expression of several genes was significantly lower in parasites growing in G6PDdeficient RBCs: TR (2.4- vs 7.3-fold), SOD-1 (3.7 vs 15.4), SOD-2 (3.7 vs 13.4), G6PD (2.6 vs 6.7), HSP60 (8.5 vs 12.9), HSP70–2 (7.5 vs 14.9). Peak expression was not significantly different in all other genes, except GR and HSP75 where peak expression was much greater in parasites growing in G6PD-deficient RBC (GR, 12.8- vs 4.4-fold; HSP75, 15.8 vs 4.8).

Comparison to parasites growing in oxidatively stressed normal RBCs was not possible, as XO/X treatment of parasitized G6PD-deficient RBCs severely impaired their viability as indicated by morphological changes and decrease in PPP flux (see below).

### Protein expression of antioxidant enzymes in parasites growing in normal and oxidatively stressed RBCs

**Figure 5:**
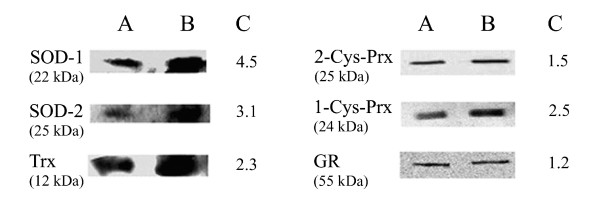
**Protein expression of antioxidant enzymes in early trophozoite stage parasites growing in normal and oxidatively stressed RBCs**. Early trophozoite stages were isolated from cultured synchronized parasites. Parasites were isolated from host RBCs by Sendai-virus treatment and lysed by freeze-thawing and sonication. Ten to 15 μg of protein extract of control (Column A) and oxidatively stressed (Column B) trophozoite stages were separated by SDS-PAGE and blotted onto nitrocellulose. The blots were hybridized with polyclonal antibodies directed against parasite superoxide dismutases (SOD-1 and SOD-2), thioredoxin reductase (Trx), thioredoxin-dependent peroxidases (1-Cys-Prx, 2-Cys-Prx), and glutathione reductase (GR), and subsequently with the secondary anti-rabbit or rat horseradish-peroxidase-coupled antibody and the bands detected by chemiluminescence and densitometric quantification. Column C shows protein expression increases in oxidatively stressed cells vs untreated controls, expressed as -fold variation of band densitometry. The results shown are representative from 1–3 independent experiments. For details, see Methods.

The effect of oxidative stress on protein expression was studied by Western blotting and band densitometry in a panel of selected antioxidant enzymes in early trophozoite stage parasites growing in normal and oxidatively stressed RBCs. As shown in Figure 5, protein expression of SOD-1, SOD-2, TR, 1-Cys-Prx, 2-Cys-Prx and GR was increased in oxidatively stressed trophozoite stages. Protein expression increases in oxidatively stressed cells vs untreated controls, displayed as change in band densitometry, were remarkable for SOD-1 (4.5-fold), SOD-2 (3.1-fold), TR (2.3-fold), 1-Cys-Prx (2.5-fold) and less evident for 2-Cys-Prx and GR (1.5-fold and 1.2-fold, respectively).

### PPP flux and G6PD enzyme activity in Sendai-virus-treated trophozoites growing in oxidatively stressed normal RBCs and in G6PD-deficient RBCs

PPP flux reflects the in vivo activity of G6PD, the first and pace-keeping enzyme of the pathway and the main producer of NADPH, the reductant utilized by the GSH- and thioredoxin-dependent antioxidant systems of the parasite. PPP flux was measured in Sendai-virus treated trophozoite-parasitized RBCs. This treatment allows measurement of the PPP flux in the parasite, since the virus lyses the RBC membrane by selectively binding to glycophorin A, leaving the parasite membrane intact and allowing elimination of cytosolic host enzymes [30,32]. Table 3 shows that compared to unstressed trophozoites, PPP flux was increased 7.1-fold in trophozoites growing in oxidatively stressed normal RBCs, and 1.9-fold in trophozoites growing in G6PDdeficient RBCs (Mediterranean variant with <5% residual enzyme activity). Additional oxidative stress by XO/X treatment decreased PPP flux in trophozoites growing in G6PD-deficient RBC, indicating parasite damage or death. Compared to unstressed trophozoites, G6PD enzyme activity measured in lysates from Sendai-virus treated and oxidatively stressed trophozoites growing in normal RBCs showed a two-fold increase but was not modified in parasites growing in G6PD-deficient RBCs.

**Table 3:** PPP flux in trophozoites growing in oxidatively stressed normal RBCs and in G6PD-deficient RBCs

	Sample	Treatment	PPP flux	-fold increase
A	Trophozoites growing in normal RBCs	nil	5.28 ± 0.84 n = 4	1

B	Trophozoites growing in oxidatively stressed normal RBCs	low XO/X b	37.4 ± 4.5 n = 3	7.1

C	Trophozoites growing in G6PDdeficient RBCs	nil	10.1 ± 1.3 n = 4	1.9

PPP flux was measured in Sendai-virus treated trophozoites growing in normal RBCs (A), oxidatively stressed RBCs (B) and G6PD-deficient RBCs (C). Trophozoite stage parasites were isolated from host RBCs by Sendai-virus treatment and incubated in RPMI 1640 without NaHCO3, but containing 1 μCi/ml D- [1-^14^C] glucose at 5% haematocrit. After 90 min of preincubation at 37°C, PPP flux was measured by quantifying produced [^14^C]-CO2 as indicated (see Methods). Production of [^14^C]-CO2 is expressed as μmol/10^10 ^cells/h at 37°C. Mean values ± SD of n separate experiments as indicated. Significance of experiments: A vs B; A vs C; and B vs C, p < 0.001 ^b^Low XO/X: XO 0.1 mU/ml, see legend to Table 2.

## Discussion

The rapidly growing and multiplying *P. falciparum *parasite generates large amounts of toxic oxidants [1,2]. Both parasite and host RBC rely on efficient antioxidant systems for survival. The parasite has two antioxidant defense systems related to the scavenger molecules reduced glutathione (GSH) [3-5] and thioredoxins [6] both kept in a predominantly reduced state by NADPH produced by G6PD, 6-phosphogluconate-dehydrogenase, glutathione reductase and thioredoxin reductase [6-9]. Plasmodium are also well adapted to survive at temperatures temporarily reaching 41°C during clinical malaria. As much as 2% of *P. falciparum *genome appears to encode HSPs expressed at various stages of the parasite life cycle [13,14]. A recent review [13] lists a total 95 genes coding for *Pf*-HSPs and HSP-related proteins, but only a small number of those has been characterized biochemically and functionally [39-43]. Several studies have analysed the response of *Pf-*HSP to hyperthermia [40,41,43-45], while response of *Pf*-HSPs to oxidative stress, extensively studied in pro- and eukaryotic cells lines [19-24], has received no attention sofar. *Pf-*HSPs of present study were selected because they were well-characterized molecules (HSP60, HSP70, HSP90 [39-43]), localized in mitochondria (HSP60 [12]) or, by analogy with other cell types, possibly responsive to oxidative stress (HSP70 [21,24], HSP90 [22]) or potential drug targets (HSP90 [42,43]).

Present work addresses two aspects of parasite defense against oxidant challenge: first, modulation of expression of antioxidant enzymes and HSPs during normal parasite development, and, second, modulation of expression of both systems in front of increased exogenous and endogenous oxidative stress, represented respectively by parasite growth under a mild and constant flux of hydrogen peroxide produced extracellularly by XO/X [34], and growth in G6PD-deficient RBCs. mRNA expression of ten *Pf *antioxidant enzymes representative of the glutathione and thioredoxin system, and five representative and possibly redox-controlled *Pf*-HSPs were analysed by qRT-PCR at early and late ring stage (13 and 16 h after reinfection), early and late trophozoite stage (19 and 28 h after reinfection) and late schizont stage (46 h after reinfection).

The results obtained with *P. falciparum *developing in normal RBCs show for the first time that both antioxidant enzymes and HSPs were expressed in a tightly correlated manner, as both systems peaked at early trophozoite stage at 19 h after reinfection, declined afterwards, and had very similar amplification factors between approx. 1.7 and 2.0 relative to mRNA expression at early ring stage. The two asymmetrical bell-shaped curves observed here appear to be functionally related to the kinetics of formation of superoxide anions, the principal generators of ROS mostly produced during the trophozoite phase when host haemoglobin is exposed to the acid environment of the food vacuole. These results basically agree with recently published *P. falciparum *transcriptome of antioxidant defense and pentose phosphate pathway enzymes in terms of time and amplitude of peak expression [46-48]. Studies have shown that the majority of expression profiles revealed a striking nonstochastic periodicity marked by a single maximum and a single minimum in relative abundance of single mRNAs [46,49]. It was also shown that concordance in time of peaking and amplification of gene transcription, i.e. peak-to-trough amplitude of the expression profile are the major signatures of functionally correlated clusters of genes [46,50]. Taken in association with the data discussed below, close temporal and quantitative correlation in expression of antioxidant enzymes and HSPs can be considered as a first evidence of functional relatedness between both classes of molecular agents. Enhanced, co-ordinated expression of antioxidant enzymes and selected HSPs discussed before was not a generalized and unspecific consequence of oxidant stress. As an example, expression of GAPDH, a crucial glycolytic enzyme, was not responsive to XO/X treatment (see Figure 2).

Response to exogenous oxidative stress was studied in parasites growing in normal RBCs subjected to a mild flow of hydrogen peroxide generated by extracellular XO/X [34]. The results confirmed the flexible and coordinated response of both antioxidant enzymes and HSPs. All mRNA species were significantly increased compared to their stage-parallel counterparts growing in unstressed RBCs, and displayed similar asymmetrical bell-shaped expression curves peaking at 19 h after reinfection and sharply declining at late schizont stage. Expression increases were not uniform, though. While most mRNAs responded with between 4 to 8-fold increases, compared to early ring expression levels, SOD-1, SOD-2, HSP60 and HSP70–2 were high responders with fold increases between 12.9 (HSP60) and 15.4 (SOD-1). Interestingly, HSP70 and HSP90 have been shown in several eukaryotic cells to be exquisitely sensitive to a variety of stimuli that increase cellular oxidation [21-24].

Protein expression of small number of antioxidant enzymes was studied by Western blotting and densitometry. Tendentially, high protein expression observed in SOD-1 and -2, Trx and Px3 corresponded to high mRNA responders, while GR was a low responder in terms of both mRNA and protein expression. Increased levels of protein expression of the 2-Cys peroxiredoxin were previously observed in parasites stressed with *tert*-butylhydroperoxide and the redox cycler juglone [51]. It should be remembered that a moderately high positive correlation was shown to exist between mRNA and semiquantitative protein abundance for each stage observed [49]. However, it is not unusual that not all mRNA levels and the respective protein levels correlate given that it was shown that post-transcriptional regulatory mechanisms control gene expression in the parasite [52,53]. On the other hand it was shown that protein abundance of several HSPs, including HSP60, HSP70 and HSP90 showed a bell-shaped curve peaking at trophozoite stage [14,49], thus corresponding to the mRNA expression pattern shown here.

Instead of measuring enzyme activities in cell lysates we measured the PPP flux in Sendai-virus treated trophozoite-parasitized RBCs. PPP flux is considered to reflect NADPH formation by the parasite more closely than G6PD activity measured in the cell lysate, due to severe restraint of G6PD activity in the cell [54]. For example, in the human RBC the maximal activity of G6PD measured in the cell lysate exceeds the basal PPP rate by over 100-fold [55]. Table 3 shows that compared to unstressed trophozoites, PPP flux was increased 7.1-fold in trophozoites growing in oxidatively stressed RBCs indicating that increase in mRNA expression of G6PD corresponded to a metabolic response. The increased flux through PPP is not surprising given that the redox balance in the parasites is likely to be affected by the stress applied. The elevated levels of hydrogen peroxide will almost certainly lead to an increased demand for NADPH to guarantee that both the thioredoxin redox system (employing TR and several peroxiredoxins) as well as the glutathione redox system (involved in maintaining an adequate intracellular GSH/GSSG ratio) are able to deal with the reduction of ROS [2-4,7].

A second model of parasite response to oxidative stress were parasites growing in G6PD-deficient RBC (Mediterranean variant with <5% residual activity). Those parasites are subjected to constantly increased endogenous oxidative stress, as G6PDdeficient RBCs have low antioxidant defense due to low steady-state levels of reduced glutathione (GSH) and severely restricted NADPH production and GSH regeneration kinetics upon oxidative challenge [56]. In addition, compartment analysis has shown extremely low levels of GSH in rings developing in G6PD-deficient RBCs [57].

Compared to parasites growing in normal RBCs, parasites growing in G6PD-deficient cells displayed distinctly increased stage-dependent mRNA expression of antioxidant enzymes and HSPs, indicating that indeed the parasite was subjected to massive oxidative stress and responded with increased expression of protective molecules. While peak-to-trough ratios between approx. 1.7 and 2 were observed in parasites developing in normal RBCs, in parasites developing in G6PD-deficient RBCs ratios between approx. 4 and 8.5 were observed in antioxidant enzymes and HSPs, with the overshooting ratios of 12.8 and 15.8 in GR and Hsp75, respectively (see Figure 3).

Parasites growing in G6PD-deficient cells displayed similar stage-dependent increases of mRNA expression in 5/10 antioxidant enzymes and 2/5 HSPs compared to parasites growing in oxidatively stressed normal RBCs (see Figure 4). Only the expression of GR and HSP 75 was significantly higher in parasites growing in G6PD-deficient RBCs, while expression of TR, SOD-1, SOD-2, G6PD, HSP60 and HSP 70–2 was significantly higher in parasites growing in oxidativeley stressed normal RBCs. Tendentially therefore, the parasite response to the endogenous (growth in G6PD-deficient RBCSs) and exogenous oxidant stress was similar but less pronounced in the former condition. It should be noted that parasite's invasion and growth have been shown to be very similar in normal and G6PD-deficient RBCs [30], while the G6PD-deficient host RBC was distinctly altered by the parasite presence particularly at ring stage [30]. In fact, the parasite has an independent GSH synthesis machinery largely independent from the host [3,5] and is apparently subjected to a less intense oxidative stress compared to the XO/X-treated parasites. The overshooting peak expression of GR might have compensatory character to ensure high levels of GSH regeneration in the parasite.

Genome-wide analysis of gene expression of cells exposed to oxidant stress has shown that the large number of affected genes are not necessarily functionally interconnected or related to the nature of the stress. The number of genes considered here was very limited compared to the total number of parasite genes and it is highly probable that many other genes are activated or repressed by the stress employed here. However, a number of elements make it likely that HSPs play a role in antioxidant defense. First, as HSPs mediate the correct refolding of denatured proteins and avoid the formation of aggregates, HSP involvement in antioxidant defense makes biological sense as ROS directly or indirectly perturb the structure of proteins. Indirect protein perturbants are for example products of lipid (per)oxidation such as 4-hydroxynonenal formed in the mature parasites [58]. Secondly, a large number of studies have documented that HSPs play a role in antioxidant defense in all eukaryotes and eukaryotic cell lines examined sofar [see for example ref. [19-24] and [59-61]]. Thirdly, a recent comparative analysis of hydrogen peroxide-induced gene expression modifications across kingdoms has shown that HSP response, notably involving HSP20, HSP70 and HSP90, was evolutionarily conserved and one protein family representing the DNAJ heat shock proteins was induced by hydrogen peroxide in all kingdoms [61]. Finally, allopurinol, a specific inhibitor of the XO/X-elicited production of hydrogen peroxide [35], abrogated increases in expression of both antioxidant enzymes and HSPs.

Studies of the effects of drugs, such as chloroquine and artemisinin, on mRNA and protein levels in *Plasmodium *suggest a certain degree of correlation between transcriptional response and alteration of protein levels. However, the amplitude of mRNA changes appears much more pronounced than alterations of protein levels observed in proteome studies [62]. Surprisingly, Prieto *et al *[62] showed that both drugs did not result in a marked up-regulation of antioxidant proteins despite the fact that both are thought to increase oxidative stress in the parasites. The latter is consistent with another study [63] showing that CQ treatment resulted in a marked elevation of protein oxidation particularly of numerous HSPs. This is not surprising as it has been previously observed in other eukaryotes subjected to oxidative stresses [12]. Concomitantly, components of the proteasome machinery are also upregulated suggesting that protein degradation might affect abundance of oxidised proteins [62,63]. These data suggest that HSP up-regulation might help directly to remove ROS and that removal of the oxidised proteins by the parasite's protein degradation machinery protects them in a more general way from the stresses they encounter. Future studies will address this possibility.

Mechanisms employed by *P. falciparum *to regulate gene expression at the mRNA level are poorly known, and putative regulatory elements have been sofar only identified *in silico *[64]. Recently, the HSP gene family was utilized as a model system to identify regulatory elements in the *P. falciparum *genome [65]. HSP genes do not appear to contain standard eukaryotic regulatory elements. Instead, a G-rich regulatory element not homologous to known eukaryotic elements was identified upstream of several *falciparum *HSP genes [65]. It is possible that the expression enhancements shown here are co-ordinated by a common mechanism controlling expression of both antioxidant enzymes and HSPs. It is intriguing to imagine that a single or few redox-dependent signaling metabolites may interact with a common G-box governing coordinated expression of antioxidant enzymes and HSPs. The ring-to-trophozoite transition is characterized by enhanced haemoglobin catabolism [66], liberation of small amounts of haem, free iron and a number of lipid (per)oxidation products, such as 4hydroxynonenal, abundantly present in trophozoites [58,67]. Which one of those molecules is responsible alone or in combination for expression enhancement is unknown and worth exploring. Of note, 4-hydroxynonenal elicited a robust increase in HSP40 and HSP70 expression in a tumor cell line [68].

In conclusion, it has been shown that mRNA expression of parasite antioxidant enzymes and HSPs was co-ordinated and stage-dependent. Both systems were shown to be redox-responsive and manifested enhancement of co-ordinated expression in oxidatively-stressed parasites and in parasites growing in G6PD-deficient RBCs characterized by blunted antioxidant defense. Finally, as widespread and efficient anti-malarials either increase oxidant stress or inhibit antioxidant defense, present results may encourage to consider the numerous anti-HSPs molecules currently developed for anticancer therapy [69-71] as potential anti-malarials.

## Competing interests

The authors declare that they have no competing interests.

## Authors' contributions

FT, OA-N, SM and PA designed the research. OA-N, ET, PMM, SM and FT performed the experiments. GG helped with the real-time quantitative RT-PCR. FT, SM and PA examined and interpreted the data and wrote the manuscript. All authors read and approved the final manuscript.
